# Employee engagement and open service innovation: The roles of creative self-efficacy and employee innovative behaviour

**DOI:** 10.3389/fpsyg.2022.921687

**Published:** 2022-09-02

**Authors:** Xiaole Wan, Ruixin He, Guixian Zhang, Jian Zhou

**Affiliations:** ^1^Management College, Ocean University of China, Qingdao, China; ^2^Marine Development Studies Institute of OUC, Key Research Institute of Humanities and Social Sciences at Universities, Ministry of Education, Qingdao, China; ^3^Management College, Xi’an Jiaotong University, Xian, China; ^4^School of Business, Qingdao University, Qingdao, China

**Keywords:** employee engagement, creative self-efficacy, employee innovative behaviour, open service innovation, SEM

## Abstract

Improving the innovation ability of organizations is the focal point of management study. This paper puts forward that innovative self-efficacy and employees’ innovative behaviour are continuous mediating variables, and discusses the influence mechanism of employees’ involvement and open service innovation from the individual factor level. In this study, a sample of 103 employees from travel companies was used to examine the hypothesis. The results show that employee engagement is positively related to open service innovation. Innovative self-efficacy plays a completely intermediary role between employee engagement and employee innovative behaviour; Creative self-efficacy and employees’ innovative behaviour play a continuous intermediary role between employees’ engagement and open service innovation. The results of this study will eventually help enterprises to carry out service innovation behaviour.

## Introduction

Under the background of globalization, all kinds of enterprises are facing great changes in consumer demand for consumption patterns and service quality (van Riel et al., 2021). It is a dilemma to organize employees to do their jobs conscientiously or to constantly “innovate” and give play to their self-efficacy and creative work. When the quality of products or technologies is no longer the only criterion to judge the core competitiveness of enterprises, the service ability gradually shows its importance. To gain a leading position in the market, it is necessary to realize open service innovation, jump out of the productization trap, eliminate the traditional closed business model, adopt an open business model, actively cooperate with external organizations, and form innovative achievements to launch the market by integrating internal and external innovative knowledge. In this development trend, scholars proposed the concept of open service innovation. That is, by constructing an open innovation platform and integrating service innovation elements inside and outside the organization, an innovation paradigm can be transformed from inside to outside, from outside to inside and from outside to outside (West and Bogers, 2014).

As an important intangible resource in an enterprise organization, employee innovative behaviours can be finally integrated and formed into organizational innovation achievements, so many researchers explore the pre-factors affecting employee innovative behaviour at the organizational level (El-Kassar et al., 2022; Hoang et al., 2022). Compared with organizational factors, individual factors derived from employees have a more direct and root effect on employee innovative activities. Intrinsic motivation and positive working mood in individual factors can positively affect employee innovative behaviours, both of which can encourage employees to accept jobs more actively and willingly and encourage employee desire to work without external force (Mina et al., 2014; Abid et al., 2021; Li W. et al., 2022). Many researches have noted that employees with professional passion are not only beneficial to their work performance but also helpful in creating good performance and competitive advantage in enterprises (Kwon and Park, 2019). Scholars summarize enthusiasm, wisdom and adherence to this positive work behaviour and mentality when employees carry out innovative activities such as employee engagement (Bakker and Demerouti, 2008). As an individual employee factor, employee engagement not only brings rich innovation inspiration to employees but also to enterprises and organizations and improves their creativity. Although existing studies have shown that employee positive work emotions, recognition and commitment to work can promote the performance of employee innovative behaviours (Lee et al., 2016; Jung and Yoon, 2018; Nathan et al., 2019; Liu and Tong, 2022), the research on employee engagement mostly centre on exploring antecedent variables and their impact on job performance, seldom explores its impact on open service innovation (Kwon and Kim, 2020), it is hard to determine how it affects the specific mechanism of open service innovation. In addition, some studies began to focus on the important function of employee psychological motivation, and the mediating of self-efficacy gradually became a new research hotspot (Zhao et al., 2022). Creative self-efficacy describes individual confidence in carrying out innovative activities and achieving innovative results. Many empirical researches have noted that employees with higher creative self-efficacy will not avoid problems encountered in innovative work but will actively solve problems and have strong self-confidence in successfully achieving innovative results, so such employees can participate more in innovative activities (Luoh et al., 2014; Beltran-Martin et al., 2017; Cai et al., 2019; Kong et al., 2019). A high degree of engagement can make a sense of identity and participate in work, which encourages employees to be more willing to finish their work and then stimulates employees to show their ideas and creativity (Jung and Yoon, 2018).

To sum up, this study will determine the influence mechanism of employee engagement on organizational open innovation from the perspective of individual employees and make an empirical test. Following the above objective, this study explores the influencing mechanism of open service innovation from the employee level, and verifies that employees, as the main body of promoting innovation, will have an impact on open service innovation due to their individual factors, which offer an angle of view for the research open service innovation. At the same time, this paper finds that there is a continuous intermediary effect between innovative self-efficacy and employees’ innovative behaviour, which is more convincing than a single intermediary path.

## Literature review and research hypothesis

This study explores the influence mechanism of employee engagement on organizational open service innovation. Kahn (1990) proposed the conception of employee engagement, people can put their energy into cognitive, emotional, and physical work under appropriate conditions to a certain extent. Schaufeli et al. (2002) defined employee engagement as a vigorous and satisfactory functional mode, he believed dedication could continuously influence employees’ perception of their work. Shuck et al. (2017) proposed that employee engagement operates through cognitive, emotional, and behavioural maintenance, intensity and direction. Lemon (2020) uses co-creation point out that employee engagement is a balance between co-creation and functionalist approaches. Based on the above research, this study held that employee engagement is degree of employee put into work roles, which also includes cognition, emotion, and social participation involvement. Specifically, cognitive engagement is the degree to which an employee engages in his or her job role, including activating and focusing on releasing efforts to achieve goals or solve challenges. Emotional involvement is the degree to which an employee experiences positive influence in his or her work role, including activating positive emotions. Social participation is the degree of social association among the working environment and the degree of sharing common values with colleagues. Social participation also requires activating, initiating and maintaining social activities related to work and actively interacting with other people (Saks, 2006).

The concept of open innovation was proposed by Chesbrough in 2003, which refers to an innovative model that emphasizes that knowledge flows in and out of organizational boundaries purposefully and uses financial and non-financial mechanisms that conform to organizational business models (Chesbrough and Bogers, 2014). Meanwhile, Chesbrough further proposed the concept and framework of open service innovation, including all services, cooperative innovation, open innovation and business model innovation. On this basis, scholars have studied the factors affecting open service innovation, Ahmed et al. (2018) explored the impact of leadership style on open service innovation. Jasimuddin and Naqshbandi (2019) studied how knowledge-based capabilities promote inbound open innovation through absorptive capacity. de Zubielqui et al. (2019) indicated trust is related to innovation cooperation, and formal contracts are an important tool for building open innovation relationships with external institutions. And when services become an important part of the company’ s value proposition, playing the function of open service innovation get crucial (Rondi et al., 2021). In the open service innovation activities inside and outside the organization, employees actively interact with customers to increase value by breaking organizational boundaries, thereby stimulating service innovation behaviour and generating innovation performance. Scholars have pointed out that human resources can effectively motivate employee positive behaviour, so as to enhance the importance of enterprise innovation (Feng and Ma, 2020; Zhao et al., 2022). Therefore, this paper puts forward hypothesis 1:

*H1*: Employee engagement is positively correlated with open service innovation.

On this basis, next, this paper proposes two mediating variables of creative self-efficacy and employee innovative behaviour to explain how employee engagement affects organizational open innovation.

### Mediating role of creative self-efficacy

Bandura (1978) put forward the conception of self-efficacy, It is one’s faith in one’s ability to achieve a specific achievement, affected by personal behaviour and motivation, and through long-term interaction with the surrounding environment and gradually improve, not independent of environmental factors, but as a personal character to play a role. Specific activities require specific self-efficacy. When employees carry out innovative activities, individuals need to invest considerable time, resources and energy and encounter different obstacles. Therefore, scholars define innovative self-efficacy as the belief or ability that individuals can use creative methods to overcome obstacles, achieve innovative work goals and complete creative work through self-perception (Puente-Diaz, 2016; Newman et al., 2018).

Shamim & Cang found there is positive relationship among employee engagement and creative self-efficacy (Shamim et al., 2019). Kumar et al. (2022) offered that positive self-identity can have a positive impact on innovation behaviour. Therefore, when employees recognize and commit to creative roles in their work, they are confident in achieving innovation results, which will inspire personnel to partake more actively in organizational innovation activities to obtain self-efficacy. This concept of role identity is consistent with the cognitive engagement dimension in employee engagement, indicating that employee engagement and job focus can enhance their confidence in achieving innovation behaviour, namely, enhancing their creative self-efficacy. At the same time, Luu (2019) proposes that employees with harmonious work passions have a strong sense of self-support to engage in work, thereby enhancing creativity and self-efficacy. Among them, the harmonious and passionate employee characteristics are consistent with the dimension of emotional engagement in employee engagement, which shows that the positive working attitude and enthusiasm in employee engagement can also be enhanced. Therefore, this paper puts forward hypothesis 2:

*H2*: Employee engagement is positively correlated with creativity and self-efficacy.

Finally, scholars’ research shows that high creativity and self-efficacy will promote innovative work (Newman et al., 2018; Lee et al., 2019), and self-efficacy and creative self-identity will promote employees’ innovative work behaviour (Afrin et al., 2022). In the role of innovative self-efficacy, employees with enthusiasm, joy and inspiration express positive feelings and provide innovative ideas to improve service, reflecting their respect for the organization (Judge et al., 2015; Madrid and Patterson, 2022). Therefore, employees with high creativity self-efficacy have the determination and motivation to complete the task of innovation, and promote the realization of open service innovation by actively communicating with colleagues and customers. Therefore, puts forward hypotheses 3 and 4:

*H3*: Creative self-efficacy is positively correlated with open service innovation.

*H4*: Creative self-efficacy plays an intermediary role between employee engagement and open service innovation.

### Mediating role of employee innovative behaviour

Employees’ innovative behaviour is generally regarded as the foundation to promote corporate performance (Luu, 2019). Its definition mainly includes two aspects: personal characteristics and the process of achieving innovation results. On the one hand, Amabile et al. (1996) proposed that individual creativity makes individuals produce ideas, and individual innovative behaviour is based on the successful practice of these ideas, creativity is the starting point of innovation. Secondly, employee initiative is considered to be the tendency of employees to take actions to improve their work, including innovative behaviour, responsibility, voice and problem prevention (Wu and Parker, 2017). Therefore, relevant research suggests that employees’ initiative prediction depends on innovative behaviour (Kwon and Kim, 2020). On the other hand, employees’ innovative behaviour can help them find innovative solutions, which have improved organizational service processes (Karatepe et al., 2020). Akram et al. (2019) noted the creative generation phase will be greatly affected by employee motivation levels, the creative outreach phase needs stronger organizational sustain. Rank et al. (2014) and Anderson et al. (2014) noted, *inter alia*, that innovative behaviour encompasses the psychosocial process between individuals, focusing on the implementation and realization of innovative ideas. This view has also become a hot research topic in domestic academic circles. Many domestic scholars have confirmed the influence mechanism of employee innovation behaviour through empirical research (Kim and Koo, 2017; Jung and Yoon, 2018).

So, this paper defines employee innovative behaviour as follows: “employees form innovative ideas and seek support for them and finally implement them through concrete actions.” Specifically, it includes proposing innovative ideas and attempting to convince others, seeking support from new technologies or methods, making appropriate plans, and striving for funds and resources to implement innovation.

Agarwal et al. (2012) found that employees’ attitude at work is the significant element affecting innovation behaviour, this research conclusion has been widely recognized by scholars. For example, Garg and Dhar (2017) showed through empirical research that professional service personnel can show better service innovation behaviour in their work, and employee engagement can be an important force to motivate employees’ innovation behaviour, Volery and Tarabashkina (2021) proposed the impact of job centrality on innovation work behaviour, that is, employees with strong job identity can better engage in innovation behaviour and obtain higher innovation performance. Khan and Abbas (2022) also emphasized that employees’ work enthusiasm and happiness in work have a positive impact on innovation behaviour. Therefore, according to research by Garg and Dhar et al. This paper puts forward hypothesis 5:

*H5*: Employee engagement is positively correlated with employee innovative behaviour.

Open service innovation is a conscious and purposeful service innovation management process for enterprises (Chesbrough and Bogers, 2014). Although Chesbrough proposed this concept in his research on the transformation of manufacturing enterprises to service enterprises under the new situation, its connotation has always existed in the service industry research. Tajeddini and Martin (2020) noted the importance of enterprises’ human resources factors to realize service innovation through empirical analysis, and the results showed that loyal front-line employees are an important prerequisite for service innovation. Kim and Lee (2014) discovered the transformation mechanism between enterprise employees and enterprise service innovation. Specifically, through the work exchange and information exchange between employees, innovative knowledge produced a contagion effect and spillover effect within the enterprise and then enhanced the overall innovative ability within the organization through these two effects. At the same time, internal knowledge sharing and organizational support promote employees’ innovative behaviour (Zhang et al., 2021), to achieve open innovation. Therefore, enterprises can consciously guide employees to express these innovations as service innovative behaviours and promote the realization of open service innovation from the inside out. Therefore, this paper puts forward hypotheses 6 and 7:

*H6*: Employee innovative behaviour is positively correlated with open service innovation.

*H7*: Employee innovative behaviour plays an intermediary role between employee engagement and open service innovation.

### Chain mediation between creative self-efficacy and employee innovative behaviour

Tierney and Farmer (2002) noted that creative self-efficacy can effectively predict employee innovative behaviour performance and evaluate the degree of employee investment in innovative work. Gong et al. (2009) noted creative self-effective employees have strong curiosity, an adventurous spirit and creative thinking, which motivate them to engage in innovative activities. Newman et al. (2018) noted that employee creative self-efficacy can improve employee innovative ability, and the degree of improvement is regulated by the strength of entrepreneurial leadership. Teng et al. (2019) analysed the creative self-efficacy and innovative behaviour of 339 hotel employees and 89 supervisors and found that innovative self-efficacy has a significant positive influence on innovative behaviour. Bagheri et al. (2022) found that creative self-efficacy plays pivotal role in cultivating employees’ innovative behaviour. Therefore, this paper puts forward hypothesis 8:

*H8*: Creative self-efficacy is positively correlated with employee innovative behaviour.

Second, Gong et al. (2009) noted creative self-efficacy is the intermediary variable of employee learning orientation affecting their innovative behaviour, and employees with learning tendency are more likely to accumulate and master successful experience and technology so that employees have more faith in achieving innovative results, which is propitious to the formation and keeping of creative self-efficacy, thus promoting the generation of employee innovative behaviour. Kumar et al. (2022) proposed that positive self-identity can promote employees’ innovative behaviour under the intermediary mechanism of creative self-efficacy, so individual factors can influence employees’ innovative behaviour through creative self-efficacy. Combined with hypotheses 2, 5 and 8, this paper puts forward the following hypotheses 9 and 10:

*H9*: Creative self-efficacy plays an intermediary role between employee engagement and employee innovative behaviour.

*H10*: Employee innovative behaviour plays an intermediary role between creative self-efficacy and open service innovation.

Finally, based on the above assumptions, highly engaged employees are more able to participate in work roles, and are also enthusiastic and focused on innovative work, and more confident to complete innovative results, this kind of creative self-efficacy is the internal motivation for employees to achieve innovative behaviour, so that employees can invest in innovative work with greater confidence and exert their greatest innovative potential. When employees want to show more innovative behaviours, they stimulate innovative inspiration in various ways, and the innovative knowledge inside and outside the enterprise can be fully blended, especially in customer contact, employees can form ideas related to service innovation to promote open service innovation in the enterprise from the inside. Therefore, this paper puts forward hypothesis 11:

*H11*: Creative self-efficacy and employee innovative behaviour play a chain intermediary role between employee engagement and open service innovation.

**Figure 1:**
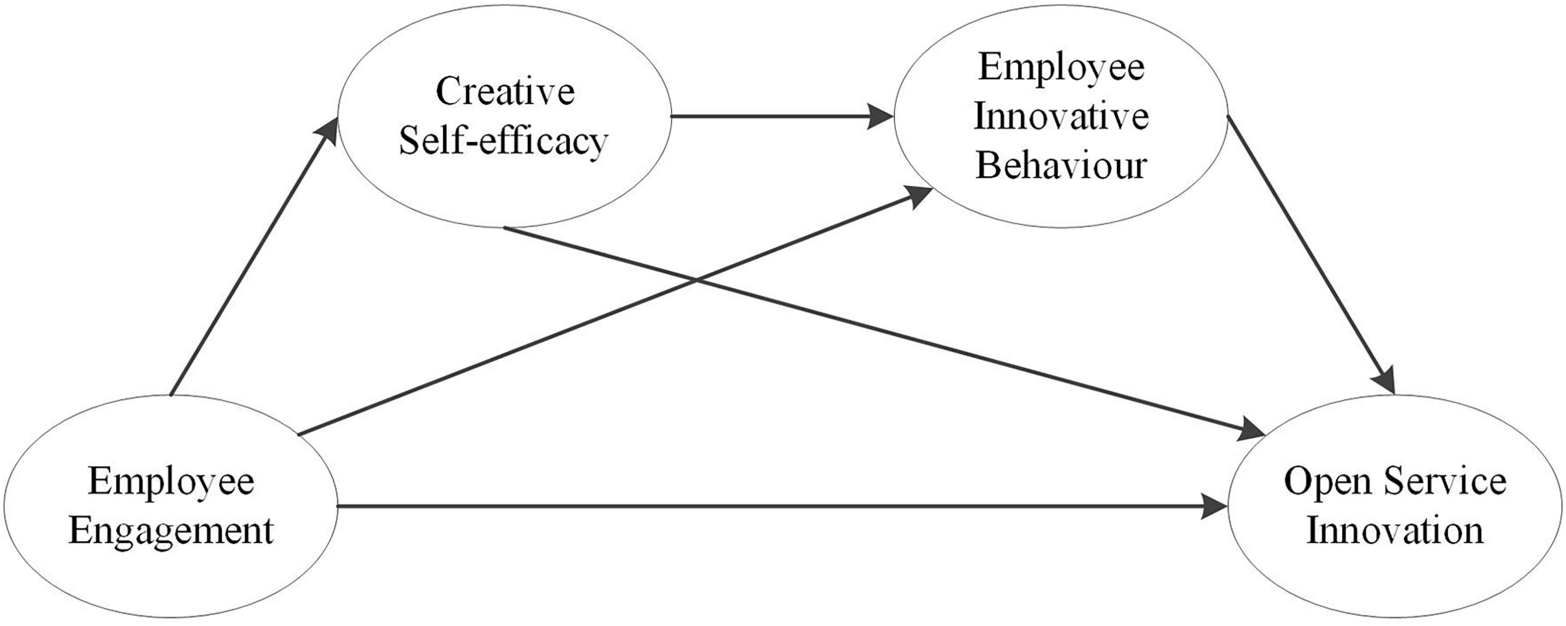
Hypothesis model of the employee engagement mechanism on open service innovation.

According to the above analysis, a hypothetical model is constructed, as shown in Figure 1.

## Research method

### Samples and data sources

This study investigates the on-the-job employees of tourism enterprises in Shandong Province. After 1 month of questionnaire distribution, collection and collation, 150 questionnaires were recycle. 103 valid questionnaires were obtained by screening, and the effective percentage was 68.7%. Descriptive statistical results of demographic characteristics of valid samples are shown in Table 1.

**Table 1:** Descriptive statistical results of population characteristics of valid samples.

Name	Category	Quantity (person)	Percentage (%)	Name	Category	Quantity (person)	Percentage (%)
**Gender**	Man	43	41.7	**Position**	Deputy general manager (member of leadership team) and above	1	1.0
	Woman	60	58.3		Deputy department manager (director, department head) and above	3	3.0
**Academic degree**	High school (technical secondary school) and below	21	20.4		Business supervisor (or project leader)	7	7.0
	Universities and colleges	46	44.7		Grassroots staff	89	89.0
	Undergraduate course	34	33.0	**Obtain employment set term of years**	Under 3 years 3–5 years 6–8 years	61 17 16	59.2 16.5 15.5
	master	2	1.9		9–10 years	3	2.9
	doctor	0	0.0		Over 10 years	6	5.8
**Enterprise scale**	Under 50 people	5	5.0	**Age**	25 years old and under	59	57.3
	50–100 people	11	10.9		26–35 years old	39	37.9
	101–200 people	10	9.9		36–45 years old	3	2.9
	201–300 people	28	27.7		46–55 years old	1	1.0
	More than 300 people	47	46.5		Over 55 years old	1	1.0

*n = 103.*

### Variable measurement

Employee innovative behaviour. According to the study results of Hu et al. (2009), the scale of five items includes proposing innovative construction, convincing others, finding new technical routes or methods, making plans for implementing creativity and striving for resources for implementing innovation. In addition, the study used a 7-point Likert scale for scoring in which 1 was totally disagree, 4 was in an intermediate state, and 7 was totally agree. The higher the score was, the more innovative behaviours the employees had. The Cronbach α coefficient of this scale was 0.883.

Employee engagement. Referring to the scale prepared by Soane et al. (2012) and Byrne et al. (2016), there were 9 items in total, including three dimensions: cognitive engagement, emotional engagement and social participation. The Cronbach α coefficient of this scale was 0.861.

Creative self-efficacy. The scale was from the research of Carmeli and Schaubroeck (2007). Based on the scale compiled by Chen (2001), they modified the basic items to closely follow the idea of creative self-efficacy. There are 8 items on the scale, which includes being able to creatively accomplish work objectives, solve work tasks, obtain work performance, and overcome work difficulties. The Cronbach α coefficient of this scale was 0.935.

Open service innovation. Referring to the research of Hu et al. (2009), there are 8 items in the scale, including the resources that the company is willing to invest in developing new services, the current human resources are sufficient to deal with the new services that need to be developed, the company provides an environment for developing new services, and the employees and departments cooperate in developing new services. The Cronbach α coefficient of this scale was 0.930.

### Pattern plan

Combined with the theoretical analysis of the four main variables and the research hypothesis, this paper establishes the following model:


(1) $$\begin{array}{l} EIB=\alpha_{1}+\alpha_{2}EE+\alpha_{3}CE\\ +\alpha_{i}Control_{gender,age,\deg ree,major,job}+\varepsilon \end{array}$$

(2) $$\begin{array}{l} SI=\alpha {'}_{1}+\alpha {'}_{2}EE+\alpha {'}_{3}CE+\alpha {'}_{4}EIB\\ +\alpha {'}_{i}{\mathrm{Control}}_{gender,age,\deg ree,major,time,job}+\varepsilon \end{array}$$

Among them, the explanatory variables are EIB (employee innovative behaviour) and SI (open service innovation), and the main explanatory variables are EE (employee engagement) and CE (creative self-efficacy). In formula (1), the coefficient $\alpha_{2}$ represents the correlation coefficient among employee engagement and employee innovative behaviour, and the expected sign is positive. Coefficient $\alpha_{3}$ represents the correlation coefficient among creative self-efficacy and employee innovative behaviour, and the expected sign is positive. Control represents a control variable. In formula (2), the coefficient $\alpha {'}_{2}$ represents the correlation coefficient among employee engagement and open service innovation, and the expected sign is positive; coefficient $\alpha {'}_{3}$ represents the correlation coefficient between creative self-efficacy and open service innovation, and the expected sign is positive; coefficient $\alpha {'}_{4}$ indicates the correlation coefficient between employee innovative behaviour and open service innovation, and the expected sign is positive. This paper analyses formula (1) and formula (2) and verifies the hypothesis through empirical research.

In order to reduce the possible method bias in this paper, in the process of data collection, the research team collected the data separately from explanatory variables as much as possible. In this paper, Harman single-factor test is also used to test the homologous variance of the main variables involved. The results show that the first factor can explain for 35.89% of the variance, and the variance can explain 74.76% of the variance. The variance explained by the first factor is less than half of the total variance. Therefore, there is no serious deviation of the common method in this paper.

## Research results

### Descriptive statistical analysis and correlation analysis

As shown in Table 2, the average values of employee engagement, creative self-efficacy, employee innovative behaviour and open service innovation are 5.59, 5.50, 5.44 and 5.53, which are still between “slight agreement” and “moderate agreement,” indicating that the surveyed employees basically agree with their employee engagement, creative self-efficacy, employee innovative behaviour and the organization’s open service innovation. In addition, a Pearson’s test was used to analyse the correlation among the main variables, and the results show except for employee engagement and innovative behaviour ( r=0.51 , p<0.01 ), employee engagement and creative self-efficacy ( r=0.73 , p<0.01 ), creative self-efficacy and innovative behaviour ( r=0.57 , p<0.01 ), creative self-efficacy and open service innovation ( r=0.59 , p<0.01 ), and employee innovative behaviour and open service innovation ( r=0.56 , p<0.01 ) also show a positive significant relationship. The analysis results preliminarily show that the hypothesis and theoretical model of this study are reasonable, but the specific mechanism between key variables needs further analysis.

**Table 2:** Mean value, standard deviation, and correlation coefficient among main variables.

Variable	1	2	3	4	5	6	7	8	9	10
1. Gender	1									
2. Age	0.19	1								
3. Education	0.21^*^	0.01	1							
4. Years of employment	0.06	0.56^**^	0.01	1						
5. Position	0.05	−0.48^**^	0.09	−0.31^**^	1					
6. Enterprise scale	−0.07	−0.23^**^	−0.10	−0.06	0.01	1				
7. Employee engagement	0.05	0.11	−0.09	0.09	0.06	0.04	1			
8. Creative self-efficacy	−0.08	0.11	−0.01	0.12	0.13	0.07	0.73^**^	1		
9. Employee innovative behaviour	−0.06	0.05	−0.02	0.04	0.11	0.01	0.51^**^	0.57^**^	1	
10. Open service innovation	−0.044	−0.040	−0.090	−0.028	0.162	0.010	0.60^**^	0.59^**^	0.56^**^	1
Average value (m)	0.58	1.50	2.17	1.80	3.84	3.66	5.59	5.50	5.44	5.53
Standard deviation (SD)	0.50	0.70	0.77	1.17	0.51	1.59	0.98	1.05	1.11	1.13

*n = 103.*

** is significantly correlated at the 0.01 level (bilateral),

* is significantly correlated at the 0.05 level (bilateral).

### Confirmatory factor analysis

This paper used AMOS24.0 for confirmatory factor analysis to test the discrimination validity of the abovementioned variables (employee engagement, creative self-efficacy, employee innovative behaviour and open service innovation), and the results are shown in Table 3. Based on the four-factor model, this study also constructs a three-factor model, two-factor model and single-factor model by combining factors. Because the sample size is less than 10 times the number of items, the items are packaged before confirmatory factor analysis, and finally, each variable contains three items. As can be seen in the table the fitting degree of each index of the four-factor model is the best compared with other models ( $\chi^{2}$ /Df = 1.862, RMSEA = 0.092, CFI = 0.961, TLI = 0.946, SRMR = 0.067), which shows that the four variables of the theoretical model proposed in this paper have better discrimination validity.

**Table 3:** Fitting indexes of four hypothetical model tests.

	*χ2*	*df*	*χ2 df*	RMSEA	CFI	TLI	SRMR
Four-factor model E	89.361	48	1.862	0.092	0.961	0.946	0.067
Three-factor model A	168.385	51	3.302	0.150	0.888	0.855	0.056
Two-factor model B	361.552	53	6.822	0.239	0.706	0.634	0.108
Single-factor model C	425.008	55	7.727	0.257	0.647	0.577	0.106

A combines EE and CE into one factor; B combine EE, CE and EIB into one factor; C combine all variables into one factor.

### Hypothesis test

Using Mplus7.0 software to analyse the sample data, this paper tests the hypothetical relationship among employee engagement, creative self-efficacy and employee innovative behaviour. The results are shown in Table 4.

**Table 4:** Analysis of hypothesis test results.

Effect relation	Hypothetical content	Estimate	SE	95% confidence interval	Significance level
Direct effect	EE → SI	0.269	1.839	[0.019,0.495]	Significant
	CE → SI	0.195	1.415	[−0.027,0.430]	Not significant
	EIB → SI	0.357	2.657	[0.169,0.357]	Significant
	EE → EIB	0.234	1.311	[−0.110,0.480]	Not significant
	CE → EIB	0.427	2.544	[0.187,0.427]	Significant
	EE → CE	0.743	11.047	[0.628,0.845]	Significant
Indirect effect	EE → SI (TOTLE)	0.341	3.068	[0.178,0.531]	Significant
	EE → CE → EIB → SI	0.113	1.626	[0.039,0.314]	Significant
	EE → EIB → SI	0.083	1.131	[−0.008,0.225]	Not significant
	EE → CE → SI	0.145	1.377	[−0.019,0.341]	Not significant
	CE → EIB → SI	0.153	1.711	[0.052,0.377]	Significant
	EE → CE → EIB	0.318	2.315	[0.130,0.593]	Significant

EE = employee engagement, CE = creative self-efficacy, EIB = employee innovative behaviour, SI = open service innovation, *n* = 103.

Table 4 shows that the path coefficient between employee engagement and creative self-efficacy is 0.743, the estimated standard error value is 11.047, and the confidence interval at the 95% level does not contain 0, indicating that employee engagement has a direct and significant positive effect on creative self-efficacy, and H2 is verified. Second, the path coefficient between creative self-efficacy and employee innovative behaviour is 0.427, the estimated standard error value is 2.544, and the confidence interval at the 95% level does not contain 0, which indicates that creative self-efficacy has a direct and significant positive effect on employee innovative behaviour, and H8 is verified.

In the direct effect analysis of employee engagement and employee innovative behaviour, the results show that the path coefficient is 0.234 (dashed line), the estimated standard error is 1.311, and the confidence interval at the 95% level contains 0, which shows that the direct effect of employee engagement on employee innovative behaviour is not significant. However, in the analysis of the indirect effect, the mechanism between employee engagement and employee innovative behaviour is tested with creative self-efficacy. The results show that the path coefficient of this path is 0.318, the estimated standard error is 2.315, and the confidence interval at the 95% level does not contain 0, which indicates that employee engagement positively affects employee innovation through creative self-efficacy.

When the intermediary variables creative self-efficacy and employee innovative behaviour join the path, the confidence interval of the direct effect of employee engagement on open service innovation at the 95% level does not contain 0, and the path coefficient is 0.269, indicating that the direct effect of employee engagement on open service innovation is significant. H1 is verified, and the total indirect effect confidence interval at the 95% level does not contain 0, and the path coefficient is 0.341, indicating that the indirect effect is significant and the mediator exists.

Among the indirect effects of employee engagement on open service innovation, there are three paths. Path one is that employee engagement affects open service innovation through creative self-efficacy. The confidence interval of the indirect effect at the 95% level contains 0, and the path coefficient is 0.145, indicating that the mediating effect of creative self-efficacy is not significant, and H4 is not valid. Path 2 is that employee engagement affects open service innovation through intermediary factors, and the confidence interval of the indirect effect at the 95% level contains 0. The path coefficient is 0.083, indicating that the intermediary effect of employee innovative behaviour is not significant, and H7 is not valid. Path 3 is that employee engagement affects open service innovation through the chain mediation of creative self-efficacy and employee engagement. The confidence interval of the indirect effect at the 95% level does not contain 0, and the path coefficient is 0.113, indicating that chain mediation is significant and H11 is verified.

In addition, the confidence interval between employee innovative behaviour and open service innovation at the 95% level does not contain 0, the path coefficient is 0.357, and H6 is verified. The confidence interval of the direct effect of creative self-efficacy on open service innovation at the 95% level contains 0, and the path coefficient is 0.195, indicating that the direct effect is not significant, but the confidence interval of the indirect effect at the 95% level does not contain 0, and the path coefficient is 0.153; thus, H3 and H10 are verified.

**Figure 2:**
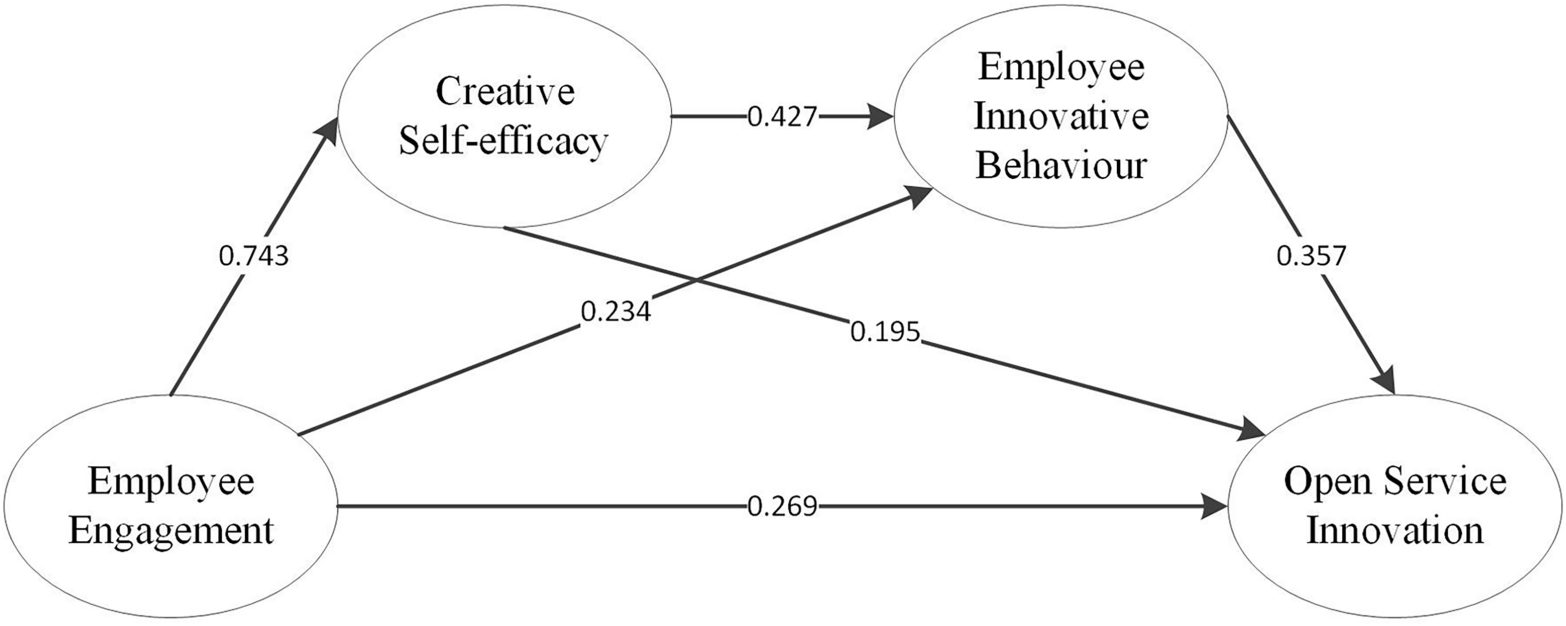
Structural equation model of the effect of employee engagement on open service innovation.

According to the above analysis, the structural equation model shown in Figure 2 is obtained in this paper.

## Discussion and conclusion

### Discussion

Facing the constant change of customer consumption concept, enterprises must break away from the traditional business model and cultivate the innovation ability of internal staff to achieve open service innovation in order to obtain sustainable competitiveness in complex markets. By collecting staff samples of tourism companies, this paper analyses and empirically tests the impact mechanism of employee engagement on organizational open service innovation, and finds that there is a continuous mediating effect between creative self-efficacy and employee innovation behaviour.

The results show employee engagement has positive correlation with creative self-efficacy. This import dedicated employees are more likely to adopt positive innovation strategies (Kwon and Kim, 2020), so employee engagement and innovation behaviour has positive correlation. Among them, employee engagement has a positive effect on innovative self-efficacy (Shamim et al., 2019), that is, when employees are more recognized for their roles and contributions at work, they tend to gain higher self-efficacy. The intensity of the relationship between the two is 0.743. therefore, employee engagement can enhance their motivation to achieve innovative behaviour, and ultimately achieve innovative behaviour, the H2 is accepted.

Second, creative self-efficacy has a positive correlation with employee innovative behaviour. Li Y. et al. (2022) used PLS-SEM to verify that self-efficacy has a significant positive impact on employee innovation behaviour, which is consistent with empirical results. Employees with higher self-efficacy have more confidence, which will encourage employees to participate more actively in innovation work, so H8 is accepted.

Third, employee engagement has no direct effect on employee innovation behaviour. Many research results show that there is an intermediate variable between employee engagement and innovation behaviour (Pan et al., 2021), that is, employee engagement positively affects employee innovation through creative self-efficacy. This result shows that creative self-efficacy as an intermediary variable can influence employee autonomy and engagement in innovation behaviour (Orth and Volmer, 2017). Xu et al. (2022) points out that more decent work can increase employee engagement, and promote employee innovation behaviour through the mediation of self-efficacy, which also supports our verification results. Therefore, H5 is rejected and H9 is accepted.

Fourthly, the path coefficient between employee’s innovative behaviour and open service innovation is 0.357, indicating there is a positive correlation between employee’s innovative behaviour and open innovation, which is logical. Employee’s innovative behaviour within the organization will promote the overall innovation of the organization, especially with the flow of knowledge resources within the organization, further promote employee’s innovative behaviour to achieve open innovation. The research of Tajeddini and Martin (2020) and Zhang et al. (2021) supports our conclusion. So, H6 is accepted. Meanwhile, the direct effect of creative self-efficacy on open service innovation is not obvious, but the indirect effect is obvious. Yang et al. (2021) and other scholars have pointed out that dual leadership at the individual level promotes open service innovation behaviour with creative self-efficacy. Our conclusion shows that creative self-efficacy first stimulates employees to realize innovation behaviour, and then promotes open innovation, which is reasonable and enriches the research in this field. Thus, H10 has been verified.

Finally, when the intermediary variable creative self-efficacy and employee innovation behaviour join the path, employee engagement has a direct correlation with open service innovation, indicating that employee engagement has a direct correlation with open service innovation. At the same time, the indirect effect is obvious, indicating that the intermediary role exists. In the three indirect influence paths of employee engagement on open service innovation, employee engagement affects the chain intermediary role of open service innovation through the chain intermediary role of creative self-efficacy and employee engagement. Therefore, H4 and H7 are rejected and H11 is accepted. This shows that employee personal factors, namely employee engagement through creative self-efficacy and employee engagement, have an impact on open innovation, which is different from the single path (Feng and Ma, 2020; Zhao et al., 2022), enriches the current study on enterprise innovation behaviour.

### Conclusion and theoretical contribution

In recent years, research on enterprise innovation, it is difficult to organize employees to strengthen self-identity to finish their work conscientiously, and it is also difficult to give full play to employees’ sense of self-efficacy and work creativity (Wang et al., 2021; Khan and Abbas, 2022). Aiming at this research problem, this paper constructs an employee engagement and creative self-efficacy mechanism model and draws relevant conclusions through empirical research data. The empirical results show that (1) employee engagement can promote open service innovation; (2) creative self-efficacy plays a complete intermediary role among employee engagement and employee innovative behaviour, and (3) creative self-efficacy and employee innovative behaviour play continuous mediating role in the relationship between employee engagement and open service innovation. These research results show that enterprises can achieve the goal of open service innovation by adopting the “conscientious” sustainable innovation development model (Ozsungur, 2020; Shin et al., 2022).

There are two main theoretical contributions from this study. First, we explore the mechanism of employee engagement in open service innovation from the employee perspective. At present, the discussion on the antecedents of open service innovation focuses on organizational rules and regulations, organizational boundaries and business models, and external users, alliances and platforms and seldom explores the influencing mechanism of open service innovation at the employee level. Therefore, this paper proposed that employees are the key subjects in promoting innovative behaviour (Kim et al., 2021), and their cognition, attitude and behaviour can affect open service innovation through contagion effects and spillover effects (Franco and Landini, 2022), thus providing a new perspective for research on the related mechanism of open service innovation.

Second, we found a continuous mediating effect of creative self-efficacy and employee innovative behaviour. Among the existing studies on the relationship among open service innovation and its antecedents, most studies build a single intermediary theoretical model, and few introduce multi-intermediary factors to deeply explore the mechanism of open service innovation. Among the three paths of employee engagement to open service innovation proposed in this paper, the indirect effects of creative self-efficacy and employee innovative behaviour as intermediary factors are not significant. Only when both are intermediary factors are the indirect effects of employee engagement to open service innovation significant. This continuous intermediary path reveals a more complex mechanism, which can reflect the specific process of employee engagement affecting open service innovation more specifically (Ul Hameed et al., 2021). Compared with the single intermediary path, it is more comprehensive and convincing and provides a theoretical basis for employee engagement and open service innovation research.

### Implications of the study for practice

Developing open service innovation is an important way to improve organizational innovation ability. This study found the mechanism of employee engagement in organizational open innovation, and next we propose recommend for the daily management of enterprises.

First, cultivate and improve employee engagement, enhance employee self-identity. Enterprise managers should uphold the people-oriented management concept, establish a positive working environment, strengthen the work centrality of employees, and implement effective human resource management methods based on respecting and understanding employees. Second, managers should try to choose employees who agree with corporate culture and corporate values, and implement them in the human resource management process of “selection, use, education, retention.” Create an equal and cooperative internal environment, promote employees to help each other in a mutually beneficial environment, help employees to devote themselves to their work role, improve the internal drive to complete the work, stimulate employees’ innovative inspiration and put into practice. Third, enterprises should pay attention to employees’ creativity and self-efficacy, and promote employees to change into reform old and innovation behaviour. Managers can provide financial, technical and other resource support for employees to carry out innovation activities, maintain a fair and just competitive environment, give employees full trust and authorization, and provide confidence for employees to complete innovation results, so as to improve the internal motivation level of employees’ competency and innovation work, and promote the generation of innovation behaviour. Fourth, encourage communication and interaction among employees. Innovative knowledge has typical contagion and spillover effects, and internal communication and interaction among employees will significantly enhance these two effects, so that employees can more effectively achieve innovative results, and ultimately provide support for promoting open service innovation in enterprises.

### Limitations and future research

First, this paper investigated employee engagement, creative self-efficacy, employee innovative behaviour and enterprise open service innovation in many tourism enterprises in Shandong Province and screened out 103 valid sample data points, so the sample sources were concentrated in Shandong Province, and all of them were employees in the service industry without complete random sampling. The analysis results may have some deviations due to the geographical situation, the particularity of different industries, different levels and types of employees. In the future, the sample range can be expanded to enterprises in different parts of the country for empirical research, and the situations of different industries can also be compared and analysed.

Second, in the aspect of variable measurement, the measurement tools proposed by foreign research scholars represented by Hu et al. (2009) may not fully reflect the specific enterprise open service innovation situation, such as manager and customer evaluation, which leads to a certain deviation between the obtained data and the actual enterprise open service innovation situation. It is necessary to improve this measurement method in terms of enterprise openness in future research.

Third, in terms of research content, the action path of employee innovative behaviour and open service innovation is not limited to the one proposed in this paper, and the starting point of action is not only the personal factors of employees but also the internal and external organizational factors. In addition, there may be a more specific and complex mechanism between each variable. For example, the influence of employee innovative behaviour on open service innovation can be realized through the mediation or regulation of knowledge sharing, which needs further discussion in future research.

## Data availability statement

The raw data supporting the conclusions of this article will be made available by the authors, without undue reservation.

## Ethics statement

Ethical review and approval were not required for the study on human participants in accordance with the local legislation and institutional requirements. The patients/participants provided their written informed consent to participate in this study. Written informed consent was obtained from the individual(s) for the publication of any potentially identifiable images or data included in this article.

## Author contributions

XW and JZ is responsible for idea generation, manuscript writing for theoretical part, and data collection. RH is responsible for idea generation and manuscript revision. GZ is responsible for data analysis. All authors contributed to the article and approved the submitted version.

## Funding

This study is supported by National Natural Science Foundation of China (Nos. 71901199, 71874167, and 71804170); Natural Science Fund of Shandong Province of China (No. ZR2021MG043); the China Postdoctoral Science Foundation Funded Project (No. 2019 M660170); the Fundamental Research Funds for the Central Universities (No. 201913015); the Shandong Social Science Planning Project (No. 19CHYJ10); the Postdoctoral Innovation Project of Shandong Province (No. 201902019); the Special Funds of Taishan Scholars Project of Shandong Province (No. tsqn20171205). The Major Program of National Social Science Foundation of China (No. 18ZDA055).

## Conflict of interest

The authors declare that the research was conducted in the absence of any commercial or financial relationships that could be construed as a potential conflict of interest.

## Publisher’s note

All claims expressed in this article are solely those of the authors and do not necessarily represent those of their affiliated organizations, or those of the publisher, the editors and the reviewers. Any product that may be evaluated in this article, or claim that may be made by its manufacturer, is not guaranteed or endorsed by the publisher.
